# Treatment of unstable forearm fractures at the metaphyseal-diaphyseal junction in children: antegrade ESIN vs. transepiphyseal intramedullary K-wire fixation

**DOI:** 10.1007/s00068-024-02562-3

**Published:** 2024-05-31

**Authors:** Markus Dietzel, Simon Scherer, Jakob Spogis, Hans Joachim Kirschner, Jörg Fuchs, Justus Lieber

**Affiliations:** 1 Department of Pediatric Surgery and Pediatric Urology, University Children’s Hospital, Hoppe-Seyler-Strasse 3, D-72076 Tübingen, Germany; 2 Department of Diagnostic Radiology, University Hospital, Hoppe-Seyler-Strasse 3, D-72076 Tübingen, Germany

**Keywords:** Pediatric trauma, Forearm fracture, Metaphyseal-diaphyseal junction, Diametaphysis, Elastic stable intramedullary nailing, Antegrade ESIN, K-wire

## Abstract

**Background:**

Treatment of unstable forearm fractures in the metaphyseal-diaphyseal junction (MDJ) zone is still a matter of debate. Major drawbacks of all types of fixations include either invasiveness, technical impracticality, or lack of acceptance by patients. This study reports results after antegrade ESIN (a-ESIN) compared to transepiphyseal intramedullary K-wire (TIK) for unstable MDJ forearm fractures.

**Methods:**

The MDJ of the forearm was defined as the square over the joints of both forearm bones subtracted with the square over the metaphysis of the radius alone. The data of 40 consecutive patients < 16 years of age who were treated either by a-ESIN (later treatment period) or TIK (early treatment period) for an unstable MDJ forearm fracture at a single high-volume pediatric trauma center were retrospectively analyzed.

**Results:**

The average age was slightly lower in the first group (TIK = 7.42 years; a-ESIN = 10.5 years). An additional ulna fracture was found in 50% of cases and was treated with a classic antegrade ESIN in 10/20 (TIK) and 6/20 cases (a-ESIN). Additional plaster cast immobilization was performed in all cases with TIK and in three cases with a-ESIN. After TIK, no complication, malalignment, or functional limitation occurred. After a-ESIN, 19/20 patients had an event-free course with stable retention and healing without axial malalignment. In one case, a temporary sensor dysfunction occurred. The same patient suffered a refracture two months after the original trauma, which required a closed reduction. Metal removal was performed after 84 days (TIK) and 150 days (a-ESIN). The outcome in all patients was good.

**Conclusion:**

Both a-ESIN and TIK are minimally invasive procedures that are technically easy to perform. Both methods are safe and lead to a complete restoration of the forearm’s range of motion. The decisive advantage of a-ESIN is the possibility of postoperative immobilization-free rehabilitation.

## Background

Fractures in childhood most frequently affect the forearm [1, 2]. Treatment of choice in unstable metaphyseal and unstable diaphyseal forearm fractures is percutaneous bi-cortical Kirschner wire fixation, and elastic stable intramedullary nailing (ESIN), respectively [3, 4]. The optimal treatment of unstable fractures of the metaphyseal-diaphyseal junction (MDJ) region between the metaphysis and the shaft is still a matter of debate [5–7]. Application, effectiveness, and safety are continuously evaluated for various techniques and compared among each other [8]. Plate osteosynthesis is stable, provides excellent alignment, but is invasive [9]. External fixation is generally well tolerated in the growing age, but is very uncomfortable at the MDJ due to long consolidation times and is therefore reserved for special indications such as multi-fragmentary fractures or refractures [10].

The above-mentioned classic approaches have been modified as alternatives in order to achieve the advantages of minimally invasive techniques. These include transepiphyseal intramedullary K-wire (TIK) fixation [7]– which provides a minimally invasive procedure, but needs additional cast immobilization– as well as retrograde ESIN using either dorsal insertion at Lister’s tubercle [11] or technical modifications such as pre-bending [6, 12] or double pre-bending [13]. However, the major drawbacks of all types of fixation include either their invasiveness, technical impracticality, or lack of acceptance by patients. Antegrade ESIN (a-ESIN) is another alternative that has always been avoided due to a high risk of injury to the posterior interosseous nerve (PIN). Nevertheless, antegrade nailing of the radius has been described for more than 20 years in adults [14, 15] with different implant entry points [16]. The posterolateral approach described by Thompson has proven its suitability for osteosynthetic fixation of distal forearm fractures [17]. In this study, patients in the growing age group were treated with a-ESIN for unstable MDJ forearm fractures and the collective was then compared to a consecutive TIK cohort.

## Patients and methods

Children and adolescents with MDJ forearm fractures which were treated using antegrade ESIN at the author’s institution were retrospectively analyzed. These were then compared to an equivalent group of patients with MDJ fractures treated with transepiphyseal intramedullary K-wire (TIK)– a technique used at the same department prior to the implementation of antegrade ESIN (a-ESIN).

Demographic characteristics, clinical background, indication for operation, intraoperative findings, and postoperative outcomes were collected from hospital records and stored on a computerized database. Endpoints were technical feasibility, stability of fracture retention and– directly related to it– the final result of fracture reduction and osteosynthesis. Data were acquired and processed according to the latest version of the “World Medical Association Declaration of Helsinki– Ethical Principles for Medical Research Involving Human Subjects”. The study was approved by the local ethical committee (project no. 630/2019BO2).

**Fig. 1:**
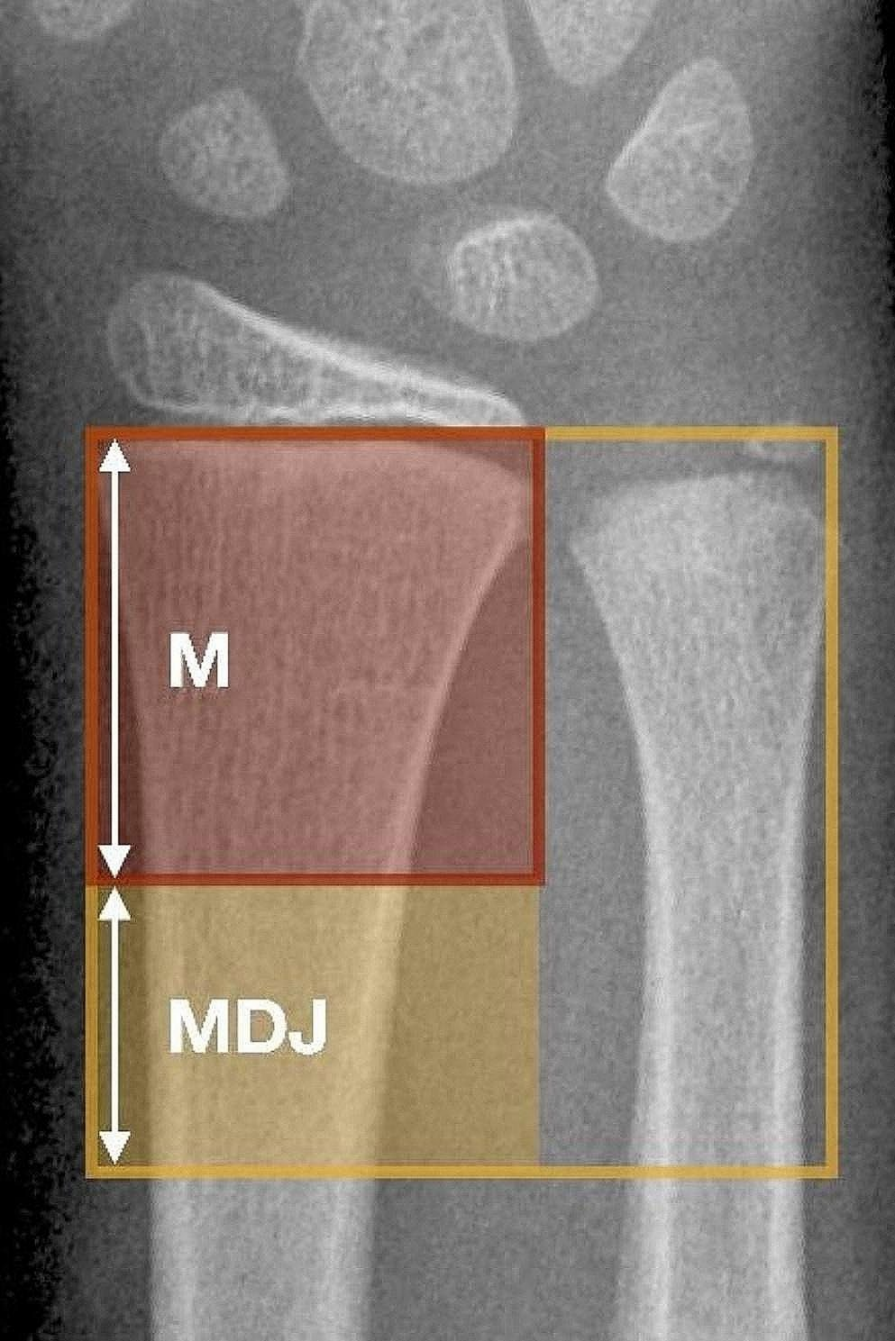
Definition of the metaphyseal diaphyseal junction area. For this study, the authors used the square over both radius and ulna and subtracted the square over the radial growth plate alone to define the metaphyseal diaphyseal transition area and to have a reproducible basis for evaluation. M = metaphysis of the radius. MDJ = metaphyseal-diaphyseal junction

In the AO Paediatric Comprehensive Classification of Long-Bone Fractures (PCCF), the metaphysis is defined as the square over the widest part of the physis of both bones on the anterior–posterior radiographic view [18]. However, a specific definition of the critical MDJ zone is not mentioned in this classification. For this study, the authors used the AO definition of the metaphysis and subtracted the square over the radial growth plate alone to define the proximal transition area and to have a reproducible basis for evaluation (Fig. 1).

Surgical intervention was indicated for one of the following two occasions: (1) when the radius was completely displaced and (2) when the radius showed a 20-degree angulation or greater while closed reduction alone could not establish stable retention.

## Results

### Patient data and fracture characteristics

From 09/2021 until 06/2023, 20 patients with unstable MDJ forearm fractures were operated on in the authors’ department using a-ESIN. For comparison of clinical results, a consecutive cohort of 20 children with unstable MDJ forearm fractures treated with TIK in previously 09/2021 were retrospectively analysed (Table 1). Low-velocity falls accounted for the majority of accidents in both groups. Fractures of the ulna were treated by classic ESIN in both groups when overall stability was affected (e.g., complete displacement/greenstick fracture; no nailing of buckle fractures or avulsion of the styloid process of the ulna). No limitation of peripheral circulation, motor function or sensitivity were recorded at the time of admission.

**Table 1:** Patients and treatment

Parameter	TIK	a-ESIN
n=	20	20
Age (years)	7.42 [4–12]	10.5 [6–15]
Ulna fracture (n)	10	10
ESIN of the ulna (n)	10	6
Cast immobilization (n)	20	3^$^
Duration of cast immobilization (days)	32.61 [21–44]	24.67 [14–32]
Time to implant removal (days)	84.54 [44–160]	150 [118–189]
Complications (n)	-	1^§^
Follow-up interval (months)	5.21 [0.9–25]	4.27 [2–5.6]
Outcome	Good^†^	Good^†^

TIK = Transepiphyseal intramedullary K-wire

a-ESIN = Antegrade ESIN

^$^ Upon surgeon’s preference

^§^ Temporary sensor malfunction

^†^ Good = Full restoration of forearm movement, no sensory or circulatory disorder at last follow-up

### Surgery details

Surgery was indicated according to the above-mentioned criteria and was performed under general anaesthesia in all cases. For the earlier group, TIK fixation was applied as described previously [7].

**Fig. 2:**
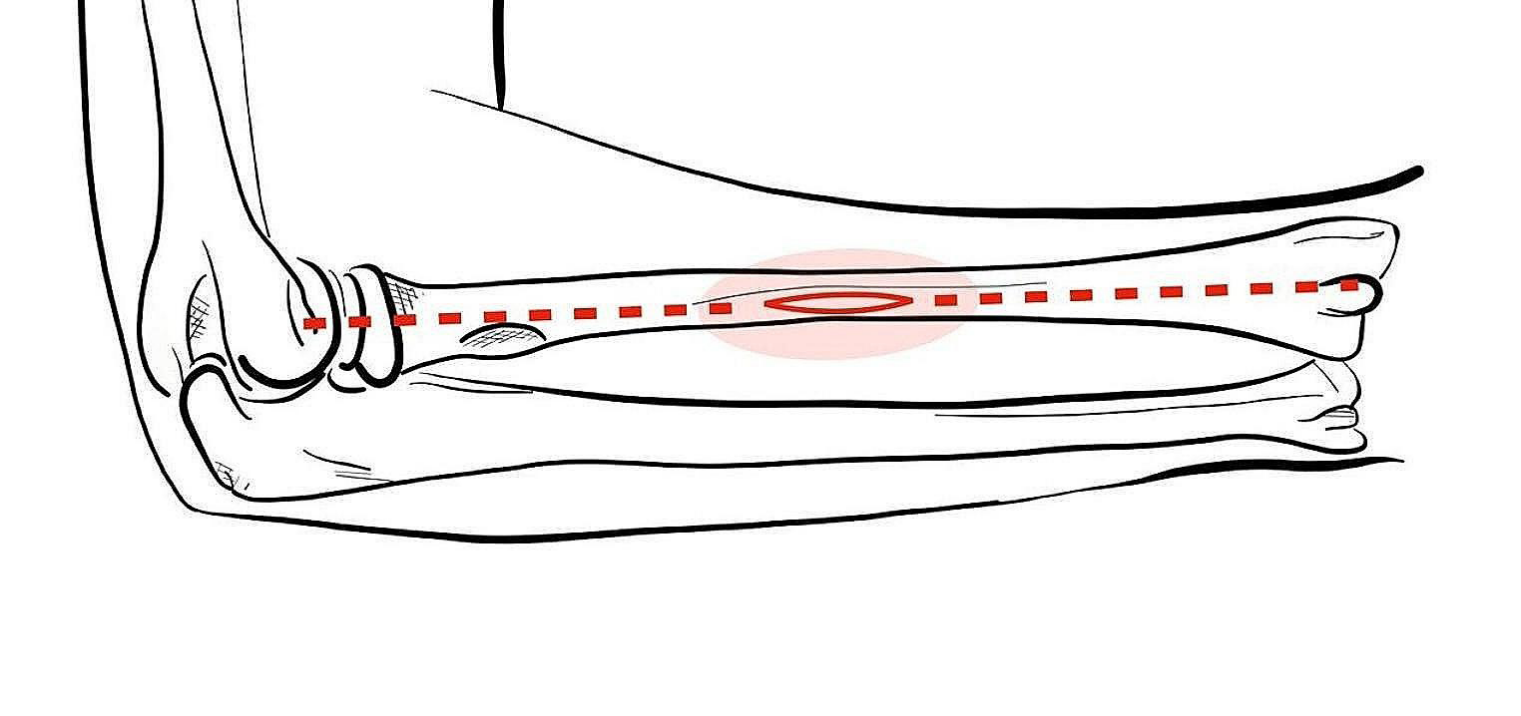
Thompson’s approach. Approach to the radius as proposed by Thompson in 1918. Landmarks are (proximal) the lateral epicondyle of the humerus and (distal) the dorsoradial (Lister’s) tubercle. In the middle of a line connecting these landmarks, a muscular “soft spot” can be identified and used for an inch-long incision for antegrade ESIN

For a-ESIN, a modified Thompson’s approach using the “soft spot” in the middle of a line connecting Lister’s tubercle to the lateral humeral epicondyle was used for nail implantation (Fig. 2). Following an inch-long skin incision, muscles were slightly retracted to reach the bone surface without greater soft tissue affection. Conventional insertion using the awl to open the diaphyseal cortex of the radius was performed in most cases and predrilling the bone with a machine-driven 2.0 mm Kirschner wire before applying the awl and then manually inserting the elastic nail was used in the latter half of the time period. No other methods of osteosynthesis were used for the definitive treatment of these fractures. Plaster cast immobilization was applied according to Table 1.

### Course and follow-up

Average hospital length-of-stay was 1.1 nights (day surgery in one case, prolonged intravenous antibiotics, and wound monitoring after open fractures in two cases each). With hospital discharge, all patients without plaster cast immobilization were granted spontaneous mobilization. All patients were planned for a follow-up visit one month after surgery and were then allowed extended everyday usage of the affected arm (e.g. bicycle riding). Metal removal was indicated when complete consolidation had been documented radiographically using X-ray controls in anterior-posterior and sagittal planes. All implants were removed under general anaesthesia in a day-surgery setting.

In the TIK group, no complications were found. In one case of a 10-year-old, motor-sensory PIN dysfunction during the first days after antegrade ESIN was reported at the first control one month following surgery. An inaccurate choice of the implant site (“soft spot”) as a possible cause was held accountable by the executing surgeons. The same patient sustained a refracture due to an ordinary fall two months after the first treatment. Closed reduction without new osteosynthesis was performed.

## Discussion

The main finding of this study is that a-ESIN is a safe and effective treatment option for unstable MDJ forearm fractures in children. The technique has long been avoided due to its proximity to the radial nerve branch during nail implantation. However, the procedure has been successfully described in adults and the surgical approach was outlined by Thompson in 1918 [17]. It provides good exposure of the radial shaft and is easy to identify during operation: The skin incision is placed on a line connecting the landmarks lateral humeral epicondyle and Lister’s tubercle at the distal radius.

Recently, Du and Han described a new operative approach, introducing an antegrade elastic nail at the “safe zone” of the proximal radius [19]. To address the gap in the literature describing an optimal entry site for antegrade ESIN to avoid posterior interosseous radial nerve (PIN) complications, Lam et al. performed a cadaveric study [16]. They concluded that if the entry point for the antegrade nail stays in between the base of the radial head and radial tuberosity with the elbow flexed and the forearm in full pronation, the entry point will be well proximal to the PIN in pediatric patients. However, here the soft tissue is thicker and nail implantation more difficult. In addition, the nail does not have to pass through the entire radius and the osteosynthesis is not a typical three-point support as described for diaphyseal fractures [3]. Du and Han also described, that they were unable to stabilize and maintain the reduction of MDJ fractures when using the Thompson approach. The problem with antegrade ESIN– regardless of proximal or more central diaphyseal nail insertion– is that the nail runs out in the wide metaphysis, that is prone to implant loosening or rather displacement of the distal radial fragment on the implant. However, this seems to be a problem with metaphyseal fractures, but not in MDJ fractures. The present study describes sufficient stability, and no secondary displacement occurred even though the majority of patients was postoperatively treated without additional immobilization. To overcome the challenge of the wide metaphyseal cavity with its spongy bone, the authors sharpened the ESIN tip with a string trimmer and then anchored the ESIN into the denser bone towards the growth plate or even slightly into it. No relevant growth plate injuries and growth disturbances occurred in this series, although the average follow-up interval of 4.27 months is still short. However, no growth disturbances have been reported after TIK either, and small bridges across the physis are considered to resolve spontaneously as a result of further growth. This has been verified by Smith et al. using MRI evaluation of the physeal region 3 weeks after transepiphyseal pinning of displaced or unstable physeal or juxtaphyseal fractures in children and 6 months after metal removal [20]. The authors describe expectable pin tracts at the 3-week time point and considerable fibrous ingrowth in the pin tracts at 6 months, but no evidence of the bony bridge, growth arrest, or any physeal abnormality. They conclude that pinning with a temporary wire across an open physis does not necessarily cause physeal growth disruption, but growth arrest or physeal damage is likely related to the original impact or injury. This is in accordance with literature reviews and case reports, which considered premature physeal arrest of the distal radius to be a rare complication after intraarticular fractures, but not after temporary K-wire fixation [21, 22]. Pin loosening or protrusion of sharp nail tips into the wrist joint have also not been observed in this series.

**Fig. 3:**
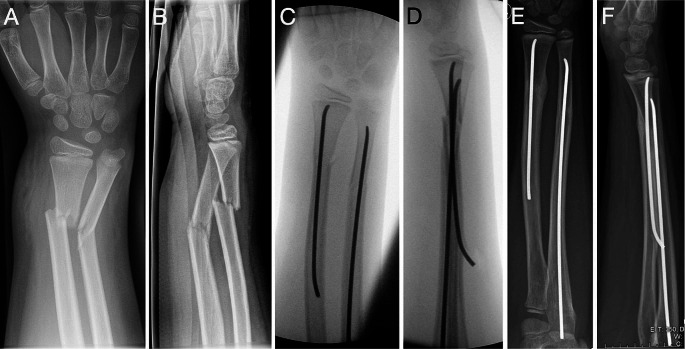
Antegrade ESIN in a 10-year-old boy. Antegrade ESIN in a 10-year-old boy. X-ray at admission (**A**, **B**), during surgery (**C**, **D**) and before metal removal after 5 months (**E**, **F**)

Of course, overall stability can only be achieved if the ulna is unfractured and forms a stable structure with the interosseous membrane. Otherwise, an unstable fracture of the ulna is stabilized by classic ESIN (Fig. 3) [23, 24]. Finally, another advantage of a-ESIN is the ability to manipulate and reduce the distal fragment by rotating the nail, similar to ESIN in radial neck fractures [25]. Also, a-ESIN avoids unnecessary dissection and disruption of the first and second extensor compartments that may occur with retrograde nailing [6, 26]. On the other hand, the diaphyseal cortex of the radius can be difficult to perforate with the manual awl, making it difficult to place an ESIN in this area without slipping off the cortex and damaging the surrounding soft tissue. Perforating the cortex with a drill or at least denting it with a machine-driven K-wire before insertion of the ESIN is a preventive measure. The authors regularly opted for this gimmick and can report simple execution. Finally, the authors did not observe skin irritation by the nail’s end, nor any restrictions of pro-/supination. However, Du and Han found some loss of forearm rotation in 10% of patients, but this resolved spontaneously after nail removal [19].

**Fig. 4:**
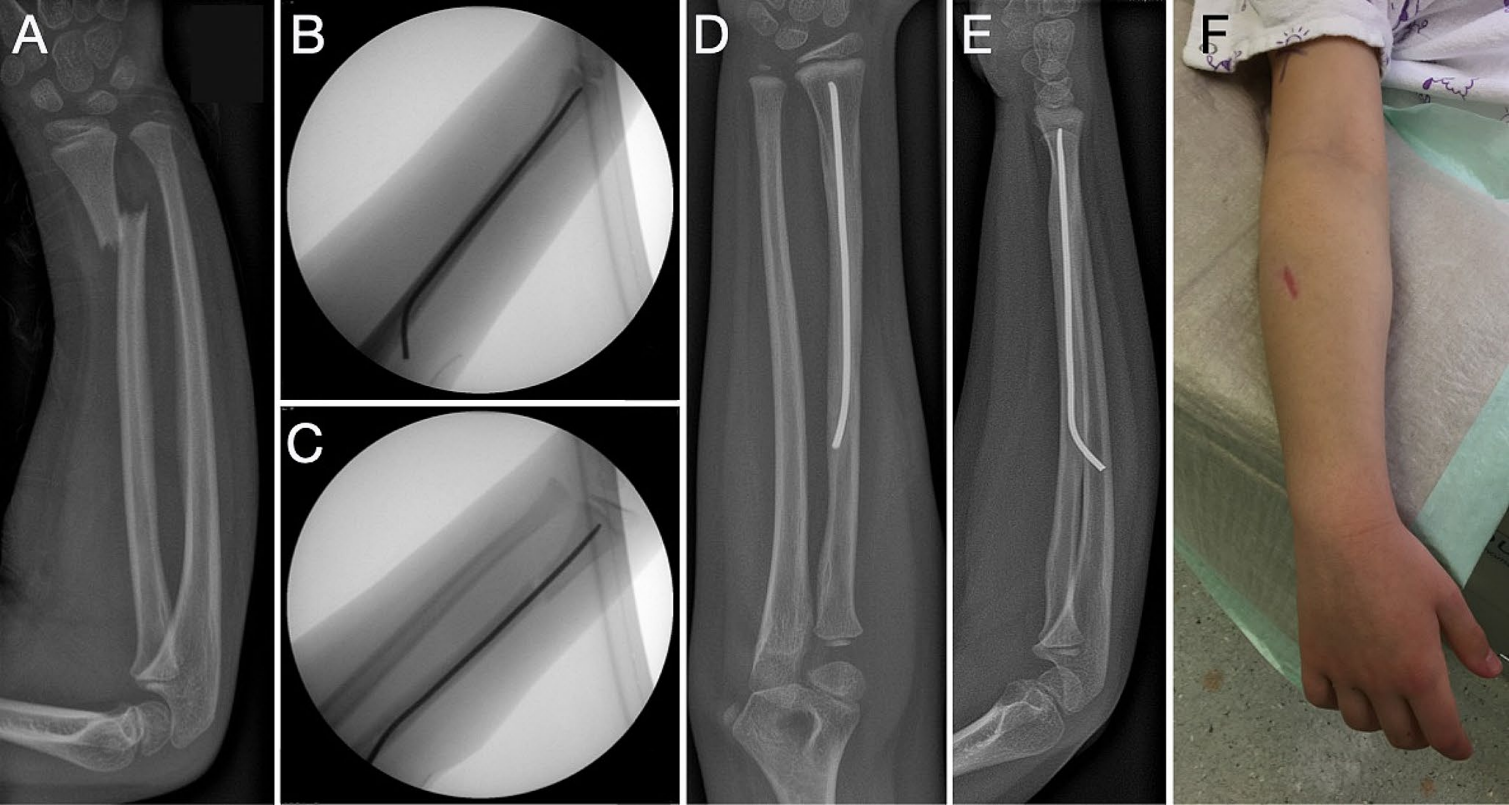
Implantation technique using Thompson’s approach. Implantation technique using Thompson’s approach in a displaced fracture located at the metaphyseal diaphyseal junction of the radius: X-ray at admission (**A**), during surgery (**B**, **C**) and before metal removal (**D**, **E**). Active scar at the dorsal aspect of the patient’s forearm following skin incision at the “soft spot” (**F**)

The clinical results in this study are encouraging. The motor-sensory disturbance of PIN reported in one case, which persisted a few days after surgery, demonstrates the importance of careful evaluation of the implantation site, using Thompson’s favorable approach. However, in this case an ultrasound scan excluded the implant to be adjacent to the nerve. In addition, reduction maneuvers and extensive nail handling may also have led to the apparent nerve involvement. Refracture with the metal in situ is a complication that cannot be influenced and has also been described in diaphyseal forearm fractures after ESIN treatment [27]. The need for anesthesia for metal removal remains a disadvantage of a-ESIN. Anesthesia for metal removal is also required for TIK, although this technique allows the wires to be left epicutaneously, which has been proven beneficial in metaphyseal fractures [28]. Finally, the cosmetic aspect of a-ESIN remains rather unattractive, as the scar is located on the visible dorsal forearm area and is noticeable when wearing short-sleeved clothing (Fig. 4). The final clinical results after both a-ESIN and TIK are comparable and very good (Table 1). However, given the advantages of antegrade ESIN, the author’s department has replaced TIK with a-ESIN as the standard of care for unstable MDJ fractures.

Even though this study showed good results, it has some limitations to mention: The patient cohort is quite small, the study is of a retrospective design, and no standardized or randomized treatment protocol was used. Nevertheless, to the author’s knowledge, this series related to MDJ fractures and a-ESIN is the most comprehensive to date. The results of this study should encourage prospective and randomized data collection, but two issues need to be addressed: (1) MDJ fractures should be included in pediatric fracture classifications to ensure a reliable means of communication for clinical interaction, education, and research. (2) Biodegradable implants are increasingly being used in pediatric traumatology and their applicability should also be evaluated in MDJ fractures [29]. E.g. Varga et al. reported that surgery with bioresorbable intramedullary implants in the treatment of severely displaced distal forearm fractures have fewer complications, reduce the number of outpatient visits and lead to significant cost savings [30]. Beyond that, further development of biodegradable implants will need to increase their stability, so that the benefits of postoperative cast-free rehabilitation can be realized. Finally, further studies are needed to better define the target population for a-ESIN in terms of age limits, fracture types and stability concerns.

## Conclusion

Antegrade ESIN in unstable forearm fractures at the metaphyseal-diaphyseal junction in children is minimally invasive, quick and technically easy, and proved to be a safe in order to achieve full recovery of forearm rotation without cosmetic deformity. Respecting the anatomical landmarks of the dorsal forearm during nail insertion makes injury to the radial nerve unlikely. This technique, performed at the radius alone or combined with ESIN of the ulna in both-bone fractures, potentially offers cast-free postoperative care and early spontaneous mobilization.

## Data Availability

No datasets were generated or analysed during the current study.

## Declarations

Karls University Tübingen (project no. 630/2019BO2).
